# Task-evoked pupillary responses track precision-weighted prediction errors and learning rate during interceptive visuomotor actions

**DOI:** 10.1038/s41598-022-26544-w

**Published:** 2022-12-21

**Authors:** D. J. Harris, T. Arthur, S. J. Vine, J. Liu, H. R. Abd Rahman, F. Han, M. R. Wilson

**Affiliations:** School of Public Health and Sport Sciences, Exeter Medical School, University of Exeter, St Luke’s Campus, Exeter, EX1 2LU UK

**Keywords:** Human behaviour, Learning algorithms

## Abstract

In this study, we examined the relationship between physiological encoding of surprise and the learning of anticipatory eye movements. Active inference portrays perception and action as interconnected inference processes, driven by the imperative to minimise the surprise of sensory observations. To examine this characterisation of oculomotor learning during a hand–eye coordination task, we tested whether anticipatory eye movements were updated in accordance with Bayesian principles and whether trial-by-trial learning rates tracked pupil dilation as a marker of ‘surprise’. Forty-four participants completed an interception task in immersive virtual reality that required them to hit bouncing balls that had either expected or unexpected bounce profiles. We recorded anticipatory eye movements known to index participants’ beliefs about likely ball bounce trajectories. By fitting a hierarchical Bayesian inference model to the trial-wise trajectories of these predictive eye movements, we were able to estimate each individual’s expectations about bounce trajectories, rates of belief updating, and precision-weighted prediction errors. We found that the task-evoked pupil response tracked prediction errors and learning rates but not beliefs about ball bounciness or environmental volatility. These findings are partially consistent with active inference accounts and shed light on how encoding of surprise may shape the control of action.

## Introduction

The idea that the brain encodes a generative model of the world to make sense of its sensory inputs has become highly influential in the fields of neuroscience and philosophy of mind^1–3^. This approach characterises the brain not as a passive recipient of information, but as an actively anticipating entity which shapes its own sensory flows. These ideas have been most extensively applied to the processing of inputs to the sensory cortices (e.g. ^4^,) but are now being extended to explain the control of actions and behaviour^5–7^. From this perspective, action (just like perception) serves to maximise the evidence for the generative model. In the current work, we examine whether the idea of the brain as a hierarchical prediction engine is consistent with oculomotor learning in an eye-hand coordination task.

The Predictive Processing Framework^1–3,8^ proposes that an organism must predict (in the broadest sense) the behaviour of its surrounding environment and the dissipative forces it presents in order to behave adaptively within its environmental niche. To this end, human brains encode a generative model representing uncertainty about hidden states of the world^9^ and perceptual and cognitive processes are driven by an imperative to minimise prediction error, i.e., the surprisal of observations^10^. During *perceptual inference* this generative model is revised by precision-weighted prediction errors, which are generated when observations deviate from expectations. The model is then updated in an approximately Bayesian fashion based on the surprisal of new observations, their perceived reliability, and the (un)certainty of prior beliefs. Subsequent work by Friston and colleagues has extended the prediction error minimisation imperative to the control of actions^5,11,12^. Under this formulation, not only can an agent minimise surprise by accurately making predictions, but they can act to change the world to minimise future surprise—a process known as *active inference*. While perceptual inference is driven by the occurrence of prediction errors, active inference behaviours, such as movement of the body or eyes, are driven by expectations of *future* uncertainty and how to minimise it (current and future uncertainty are akin to variational and expected free energy in free energy principle formulations^9^).

Expected and unexpected uncertainty are hypothesised to play a central role in adaptive learning behaviours and appear to be encoded by numerous interconnected neuromodulatory systems in the brain^13,14^. Specifically, neuro-computational learning accounts propose that under conditions of greater uncertainty, bottom-up sensory signals should be prioritised over top-down expectations, to facilitate faster response to a changing or unknown environment^13^. This equates to upweighting the neuronal gain of new sensory signals (or deviations from predictions; Friston, 2010). This neuromodulation seems to be at least partially controlled by noradrenaline, with encoding of prospective uncertainty linked to noradrenergic signals that originate in the locus coeruleus^13–17^. The effect of this upweighted signalling is a greater influence of sensory information on perception and a faster rate of belief updating^18^. There is growing evidence that activation of the noradrenergic locus coeruleus enhances sensory learning^19^, while noradrenaline blockade impairs reversal learning and cognitive flexibility^20^.

Uncertainty encoding has often been studied using pupillary dilation as an index of changes in the locus coeruleus-norepinephrine system^15,21^. Non-luminance mediated changes in pupil diameter have been shown to track the probabilistic surprise of new sensory observations^22–25^. As a result, pupil dilation has been adopted as a measure of the physiological response to prediction errors (i.e., the difference between what is occurring and what is expected) in work testing predictive coding hypotheses (e.g.^16,26^,). Given the increasing prominence of neuro-computational approaches in psychology and neuroscience research, these objective measurement techniques may help develop our understanding of how sensory information is retrieved, processed, and responded to across the central nervous system. Indeed, compared to more direct measures of neuronal prediction errors signals, such as EEG and fMRI^27–29^, pupillometry offers a less invasive alternative that holds promise for advancing this theoretical work.

To date, research examining the correspondence of task-evoked pupillary responses with probabilistic surprise has focused on associative learning and perceptual inference^21,26,30^. We sought to extend this work to explore whether similar pupillary responses could also be observed in relation to estimates of probabilistic surprise associated with *active inference* (e.g., the control of fixations and saccades by the visual system^6^). Specifically, we have tested the hypothesis that the dynamic updating of anticipatory eye movements over successive trials is related to physiological encoding of surprise by the noradrenergic system. In a previous study, Lawson et al.^16^, demonstrated the link between prediction errors and pupil size, and the role of noradrenaline in this signalling of surprise. Vincent et al.^26^ have also shown that pupil dilatation tracks not only surprise on aberrant trials but also long-term belief updating, i.e., tonic changes to the baseline pupil diameter. Further, Vincent et al.^26^ report that an ideal Bayesian observer model provided the best explanation of these tonic changes. In essence, larger dilation corresponds to both short term surprise (in the Bayesian sense of deviation from predictions rather than the emotional reaction)^25,31^ as well as longer term encoding of uncertainty about beliefs. Crucially, we tested whether these effects were also present in the context of a dynamic movement task—the manual interception of a bouncing ball performed in virtual reality (VR). We recorded a single eye movement metric that indexes predictions in this task and fitted participant-wise models of Bayesian inference to these data^32^ to estimate individual trajectories of beliefs, prediction errors, and learning rates. Finally, we examined whether pupil responses tracked (i) the parameters estimated from these active inference behaviours and (ii) parameters from a simulated optimal Bayesian observer. It was predicted that:

- *H*_*1*_: The trial-to-trial learning of anticipatory eye movements would be better explained by a hierarchical Bayesian inference model than a simple associative learning model;
- *H*_*2*_: Task-evoked pupil responses would be related to prediction errors and rate of learning during active inference;
- *H*_3_: Task-evoked pupil responses would be related to the perceived volatility of the environment;
- *H*_*4*_: Pupil responses would more closely track the parameters from the personalised active inference models than a theoretical (i.e., simulated) Bayesian observer.

## Methods

### Design

The data reported here were collected as part of a larger study examining the effect of anxiety on predictive eye movements and movement kinematics during an interceptive task. Here, we report data only from the baseline (low anxiety) conditions. Data were collapsed across two non-contingent feedback sub-conditions (both low anxiety) as the feedback occurred after the eye movements and therefore should not impact trial-to-trial changes in the task-evoked response. To mitigate against any tonic changes to pupil dilation as a result of the feedback conditions, all pupil response data were first baseline corrected and then normalised by the standard deviation (see below for more details).

### Participants

Forty-four participants (ages 18–30 years, mean = 22.8 ± 2.3; 23 males, 21 females) were recruited from the population at a UK University to take part in the study. Participants were naïve to the aims of the experiment and reported no prior experience of playing VR-based racquet sports. They attended a single session of data collection for ~ 1.5 h. No a-priori power analysis was conducted for the analyses reported here, so a sensitivity analysis was run to determine the types of effect we were powered to detect. For the one sample *t*-tests used to determine whether β coefficients were non-zero, we were able to detects effects of *d* = 0.33 with 70% power, *d* = 0.38 with 80% power, and *d* = 0.45 with 90% power (given *n* = 44 and α = 0.05). Ethical approval was provided by the School of Sport and Health Sciences Ethics Committee before data collection and all participants gave written informed consent prior to taking part. The study methods closely followed the approved procedures and the Declaration of Helsinki.

### Task

**Figure 1:**
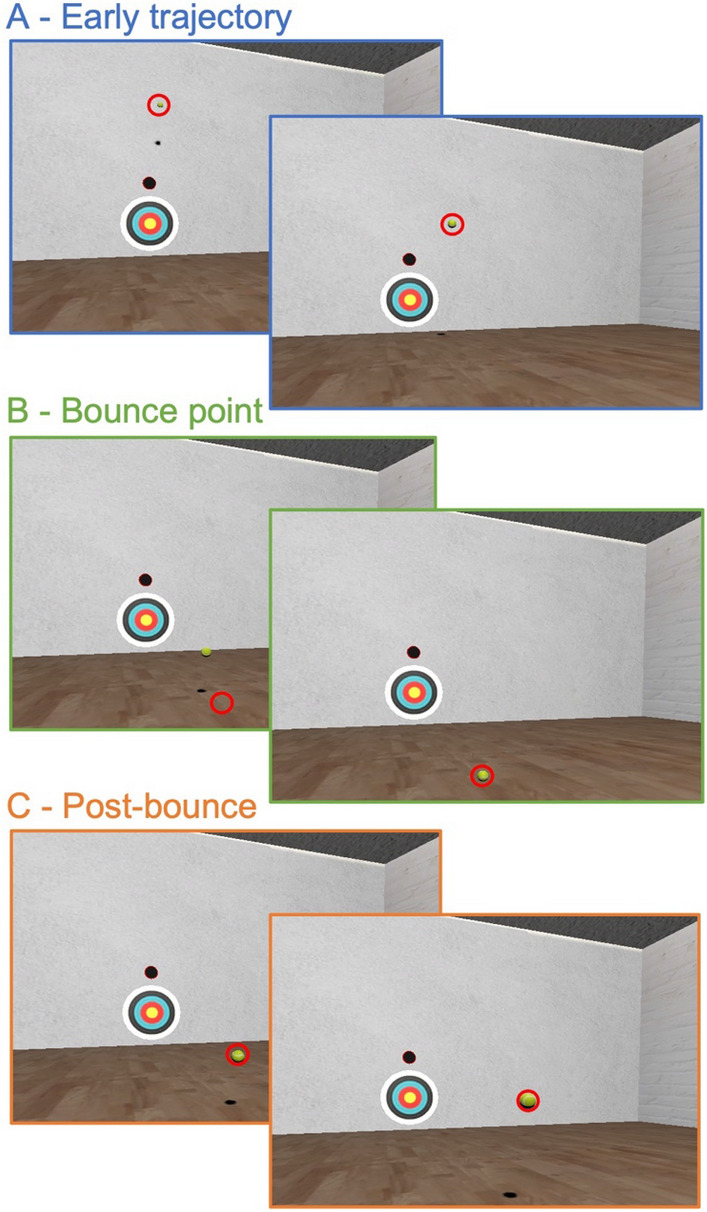
Typical interceptive eye movements in this task. Figure shows screenshots from the interception task with theoretical point of gaze (red circle) superimposed. The figure shows successive points in the ball trajectory form the early release (**A**), just before and during the bounce point (**B**), and during the post-bounce period, just before the ball is hit. Gaze typically tracks the early portion of the trajectory, then saccades ahead to the bounce point, waits for the ball to catch-up, then tracks the ball during the post-bounce portion.

**Figure 2:**
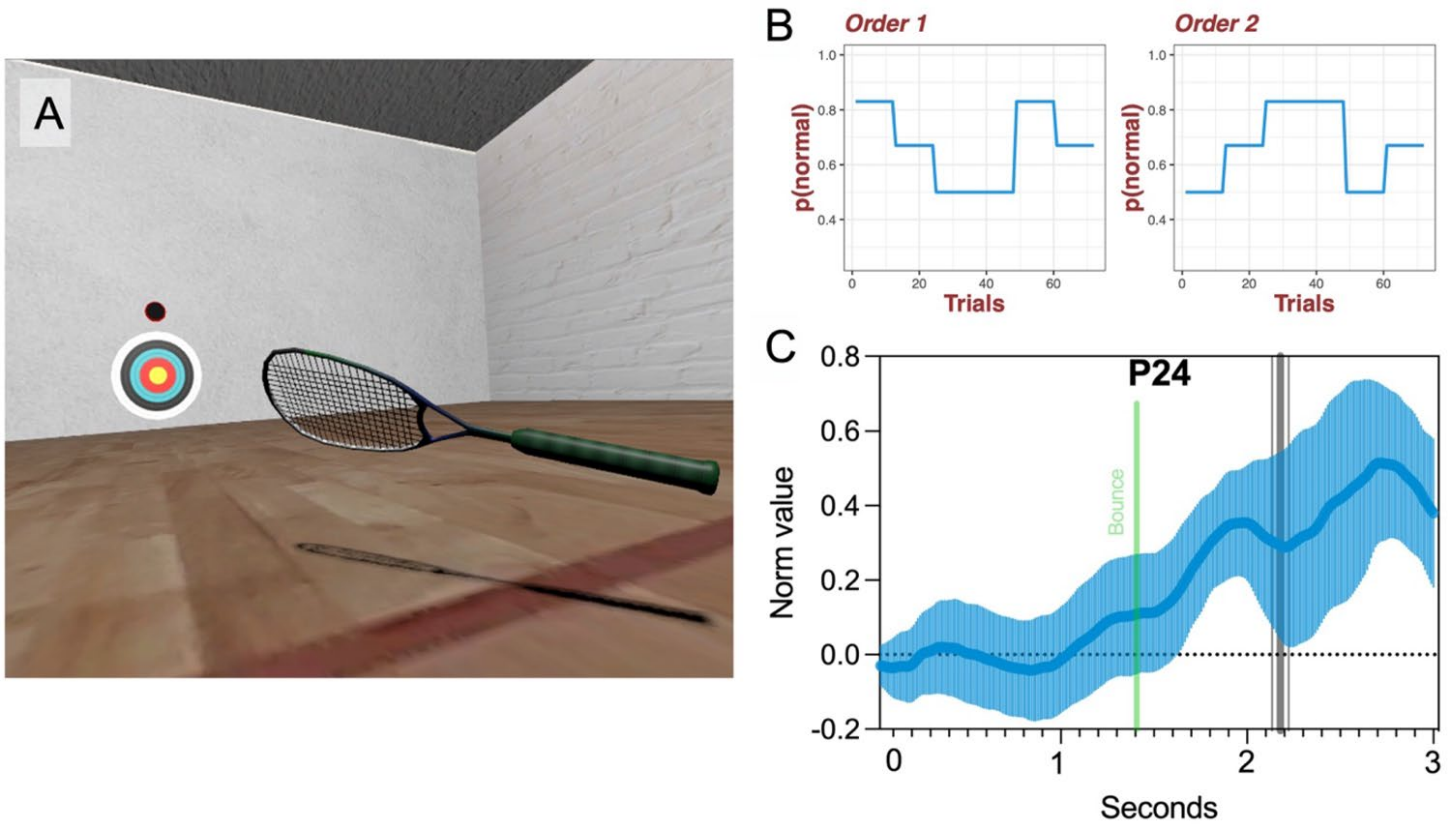
Task environment, trial orders and the pupil response. Panel A shows the VR environment and animated racquet. Panel B show the volatile trials orders where trials regularly shift between periods of *p(normal)* = 0.5; 0.67; and 0.83. Panel C illustrates task-evoked pupil responses for a single participant (P24) with the mean in bold and error bars showing the standard deviation. The bounce point (green vertical line) and mean interception point (vertical thick grey line with thin grey lines showing the standard deviation) are also marked. Plots for all participants are available in the supplementary files.

Participants performed a VR interception task previously developed by Arthur et al.^33^ to examine active inference in autism (the task code is available from the Open Science Framework: https://osf.io/ewnh9/). Participants were placed in a virtual environment that simulated an indoor racquetball court. The court (see Figs. 1 and 2A) spanned 15 m in length and width. A target consisting of a series of concentric circles was projected onto the front wall. Above this target was an additional circle (height: 2 m) where virtual balls were launched from during each trial. The floor resembled that of a traditional squash court and participants were instructed to start behind the ‘short line’ (located 9 m behind front wall, 0.75 m from the midline). The experimenter checked that participants were stood in the correct location at the start of each trial. On each trial, the ball was projected towards the participant and they were instructed to hit it back to the projected target circles using a virtual racquet, operated by the Vive hand controller. Virtual balls were 5.7 cm in diameter and had the visual appearance of a real-world tennis ball. The visible racquet in VR was 0.6 × 0.3 × 0.01 m, although its physical thickness was exaggerated by 20 cm for the detection of ball-to-racquet collisions.

The VR task (see Fig. 2A) was developed using the gaming engine Unity 2019.2.12 (Unity technologies, CA) and C#. The task was displayed through an HTC Vive Pro Eye (HTC, Taiwan) head-mounted display, a high precision VR system which has proven valid for small-area movement research tasks^34^. The Pro Eye headset is a 6-degrees of freedom, consumer-grade VR-system which allows a 360° environment and 110° field of view. Graphics were generated with an HP EliteDesk PC running Windows 10, with an Intel i7 processor and Titan V graphics card (NVIDIA Corp., Santa Clara, CA). Two ‘lighthouse’ base stations recorded movements of the headset and hand controller at 90 Hz. The headset features an inbuilt Tobii eye-tracking system, which uses binocular dark pupil tracking to monitor gaze at 120 Hz (spatial accuracy: 0.5–1.1°; latency: 10 ms, headset display resolution: 1440 × 1600 pixels per eye). Pupil diameter data were recorded by the Tobii eye-tracking system and accessed in real-time using the SRanipal SDK (see: https://developer.vive.com/resources/vive-sense/eye-and-facial-tracking-sdk/).

### Measures

#### Gaze pitch angle

Previous work has demonstrated that predictive eye movements can be used to model active inference during interception of a bouncing ball^35^. When intercepting a ball in this task, individuals have been shown to direct a single fixation to a location a few degrees above the bounce point of the oncoming ball^36,37^ (see Fig. 1). Crucially, the spatial position of this fixation (the gaze pitch angle) is sensitive to beliefs about likely ball trajectories, with fixations directed to a higher location when higher bounces are expected^36^. As the fixation occurs before the bounce is observed, the fixation location is driven by an agent's prior expectations about ball elasticity and therefore provides an indicator of the evolution of beliefs over time^35^.

The gaze pitch angle was calculated from the single unit gaze direction vector extracted from the inbuilt eye-tracking system (head-centred, egocentric coordinates). All trials were segmented from ball release until ball contact. Gaze coordinates were treated with a three-frame median smooth and a second-order 15 Hz low pass Butterworth filter^38,39^. Based on the procedures reported in Arthur et al.^33^, trials with > 20% missing data were excluded as this could indicate poor tracking, as were trials where eye-tracking was temporarily lost (> 100 ms), which could cause erroneous detection (or non-detection) of a fixation. A spatial dispersion algorithm was used to extract gaze fixations^40^, which were operationalised as portions of data where the point of gaze clustered within 3° of visual angle for a minimum duration of 100 ms^41^. After performing the fixation detection procedure, we extracted the position of the fixation that occurred immediately (< 400 ms) prior to the bounce (expressed as gaze-head pitch angle). Data values that were > 3.29 SD away from the mean were classed as outliers (*p* < 0.001), and participants with > 15% of data identified as missing and/or outliers were excluded (in line with^33^). As in Arthur and Harris^35^ the pitch angle variable was then converted to a discrete variable for modelling purposes; when the gaze angle shifted to a lower spatial location than on the previous trial (> 1 SD change) this was taken as a shift towards higher expectation of *p(normal)* and vice versa.

#### Pupil dilation

Binocular pupil diameter (in mm) was recorded at 90 Hz from the in-built eye tracking system in the VR headset. The data were processed using protocols well established in the literature and adapted from the PUPILS Matlab toolbox^42^. Firstly, blinks were identified from portions of the data where the pupil diameter was 0, before being removed, padded by 150 ms, and replaced by linear least-squares interpolation^42,43^. The resulting signal was then filtered using a low-pass Butterworth filter with 10 Hz cut-off. Right and left eye data were treated separately then averaged.

To account for fluctuations in arousal and tonic pupil changes, we performed a baseline correction, as recommended by Mathôt and Vilotijević^44^. Baselining was achieved by subtracting the baseline pupil size, taken from a 200 ms window before stimulus onset (as in^16^), from the peak pupil response over a 3000 ms window on each trial (see Fig. 2C). This duration was chosen as pupil size tends to peak around one second after stimulus onset^45^, so should be sufficiently long as to allow changes in pupil size of cognitive origin to emerge^44^. Trials were also separated by at least 2–3 s as recommended by Mathôt and Vilotijević^44^. Following Vincent et al.^26^, the data were then normalised by their standard deviation, such that the final time series represented the number of standard deviations from the mean. This enabled us to equate values across subjects, while accounting for participants with overall smaller pupillary responses due to differential sensitivity to luminance. As the VR environment provides a constant luminance level, and the scene was static apart from the projected ball, there was little to no variance in lighting from trial to trial. Trials with > 20% missing data, or where eye-tracking was temporarily lost (> 100 ms) were excluded. Data values that were > 3.29 SD away from the mean were classed as outliers (*p* < 0.001), and participants with > 15% of data identified as missing and/or outliers were excluded. One participant was removed from the outset because no pupil data were recorded due to an error and six further participants were removed for missing pupil data. Of the remaining participants, less than 5% of trials were missing (see the supplementary files for a full breakdown of missing trials).

### Procedures

On arrival at the laboratory, participants had the experimental tasks verbally explained to them and then provided written informed consent. They were fitted with the Pro Eye VR headset and the inbuilt eye-tracker was calibrated over five locations, and then recalibrated after any displacement of the headset. Participants then completed five familiarisation trials of the interception task. During each trial, individuals were instructed to hit the oncoming ball back towards the centre of the projected target. Ball projections were signalled by three auditory tones, and passed exactly through the room’s midline, bouncing 3.5 m in front of the prescribed starting position. All participants were right-handed so started 0.75 m to the left of the midline so that all shots were forehand swings. All projected balls were of identical visual appearance but had two distinct elasticity profiles—one bounced like a normal tennis ball while one had drastically increased elasticity such that it generated an unexpected post-bounce trajectory that is totally unlike a real tennis ball. The two ball types followed the same pre-bounce trajectory and speed (vertical speed: − 9 m/s at time of bounce), which was consistent with the effects of gravity (− 9.8 m/s^2^). The ball made a bounce noise when it contacted the floor and then disappeared on contact with the racquet, to prevent uncontrolled learning about elasticity between trials. Participants were told that the experimenter could still see where the ball went, but that they themselves could not.

To create conditions of high environmental volatility, we systematically varied ball elasticity over time. In *normal* (aka expected) trials, ball elasticity was congruent with its visual ‘tennis ball-like’ appearance, set at 65%. Conversely, in *bouncy* (aka unexpected) trials, elasticity was increased to 85%, to produce an abrupt change in ‘bounciness’ that is easily detectible to participants^46^. We then varied the probability of p(normal) over time, shifting between periods of 0.5, 0.67, and 0.83 to create a volatile environment. Participants completed four blocks of 72 trials, two in low anxiety conditions plus two in high anxiety conditions, which are not reported here. There were two possible order sequences which were counterbalanced across participants (see Fig. 2B). No explicit information about ball elasticity, trajectory, or probabilistic manipulations was provided.

### Computational modelling

Regressing pupil dilation onto a simulated model of Bayesian inference (e.g. ^21^,) assumes that each participant learns the ground truth of the experiment to the same extent, such that trials experienced as ‘unexpected’ to one participant ought to be ‘unexpected’ to another. By also fitting a model to the responses of each individual, we were able to characterise trial-to-trial belief updating based on the anticipatory eye movements each participant made. We could therefore characterise which observations were most ‘unexpected’ for each individual, as well as being theoretically ‘unexpected’. Computational modelling analyses therefore consisted of two elements: (i) generating an ‘optimal’ model of inference to determine where the largest prediction errors should occur *in principle,* given our trial orders and (ii) fitting personalised models to each participant’s data.

**Figure 3:**
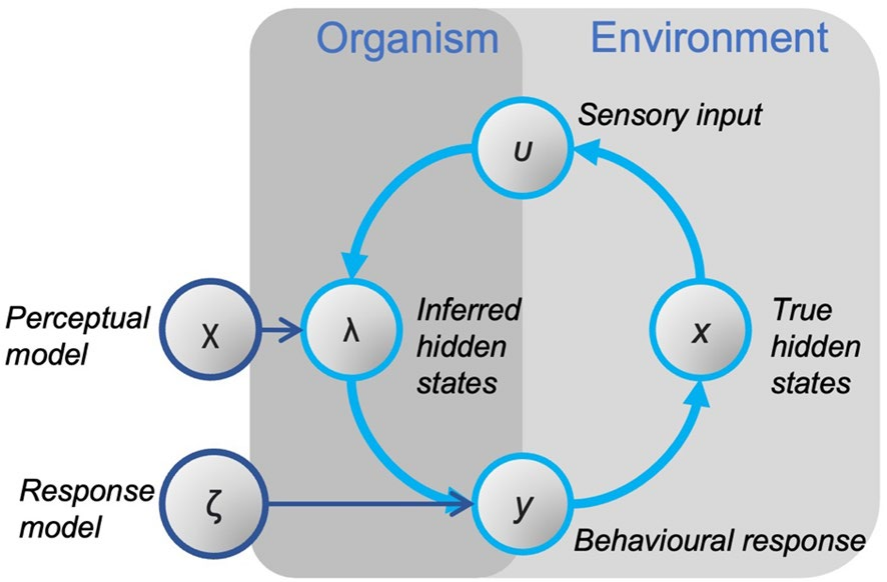
Schematic representation of basic HGF framework. Predictive processing and active inference formulations describe an agent as connected to its environment indirectly by the sensory information it receives (**u**) and the actions it takes (**y**) (i.e., blanket states). An agent must therefore perform Bayesian inference to generate an estimate of the true hidden state of the world (**x**) based on sensory input. In the HGF, the evolution of these estimates over time are described by the perceptual model (**χ**). The responses the agent makes depends on beliefs encoded in the perceptual model, and the relationship between beliefs and behavioural responses are described by the response model (**ζ**).

**Figure 4:**
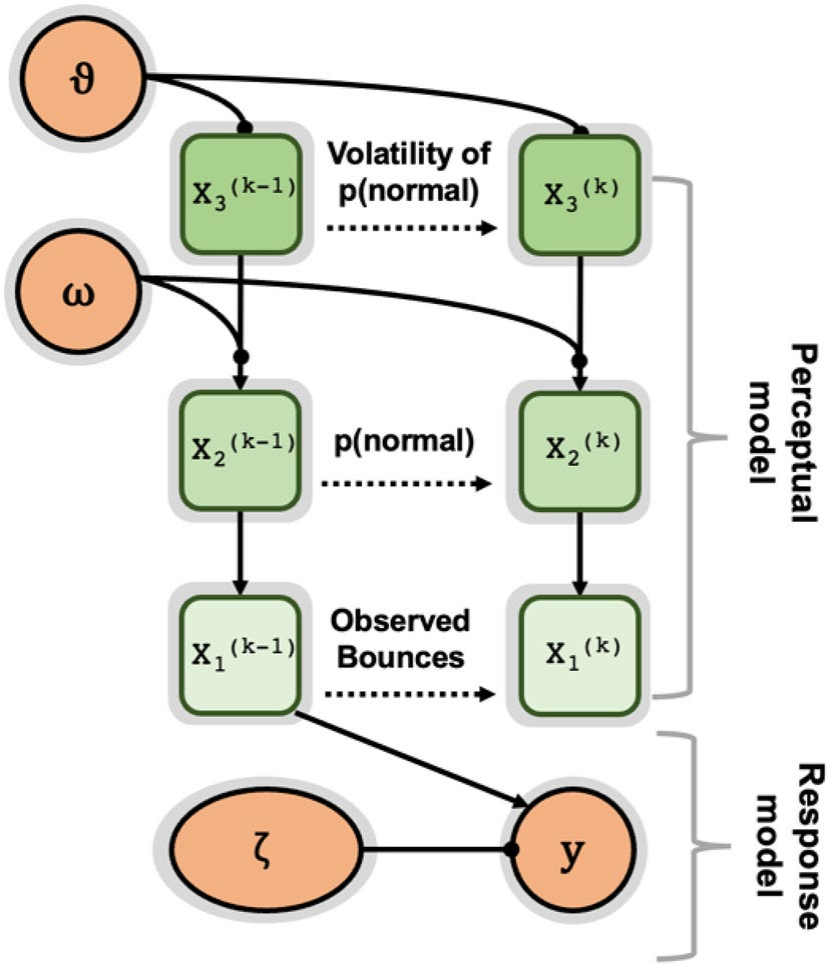
Schematic of three level HGF model. Level x_3_ corresponds to perceived volatility of the evolving beliefs about the probability of normal/bouncy at x_2_. The relationship between beliefs at x_2_ and decisions over action are described by the sigmoid transformation of x_2_ at x_1_. Parameters $\mathrm{\vartheta }$ and ω determine the variance in the Gaussian random walk for their respective levels. In the absence of perceptual uncertainty, as is the case here, x_1_ simply corresponds with observations (u). Time steps are denoted by k.

For both approaches, we used the Hierarchical Gaussian Filter (HGF) model of perceptual inference^32,47^, a modelling approach that has been used widely to model tasks like associative learning under uncertainty^48,49^. The HGF adopts a framework where an agent receives a time series of inputs to which it reacts by emitting a time series of responses (see^32,47^; Fig. 3). The model assumes Gaussian random walks of states at multiple levels where the variance in the walk is determined by beliefs at the next highest level (see Fig. 4). The coupling between levels is controlled by parameters that shape the influence of uncertainty on learning in a subject-specific fashion. The Bayesian inference process is modelled via a *perceptual model,* which describes the core inference process of belief updating from on observations, and a *response model*, which describes how beliefs translate into decisions to act (see Fig. 3). Crucially, when both inputs (observed ball bounces) and responses (anticipatory eye movements) are known, the parameters of the perceptual and the response models can be estimated.

While previous work has supported hierarchically-ordered perceptual learning^50,51^, we also examined whether participants' active inference behaviours could instead be explained by simpler non-hierarchical models, like traditional reinforcement learning^52^. We therefore compared two hierarchical models—a 3-level HGF and a 4-level HGF—with a simple Rescorla-Wagner (R-W) learning rate model. Reinforcement learning models postulate that agents learn to take actions that maximise the probability of future rewards^52^. Predictions about a value (*v*) are updated over trials (*k*) in proportion to the size of the preceding prediction error (δ) and a stable learning rate scalar (α):

$$ \Delta v^{k} \propto \alpha \delta^{k} $$

The R-W model fundamentally differs from Bayesian learning models (e.g., the HGF and partially-observable Markov decision models^53^;) as learning rates are fixed and do not evolve based on hierarchical estimates of parameter changeability. Hence, the impact of the prediction error is entirely dependent on the size of the error, rather than flexible precision-weighting based on the strength of priors or likelihoods.

The open source software package TAPAS (available at http://www.translationalneuromodeling.org/tapas;)^54^ and the HGF toolbox^32,47^ were used for model fitting and comparison. Additional details of the mechanics of the model are described in the supplementary files (see: https://osf.io/z96q8/) and in Mathys et al.^32^.

By fitting the parameters of the perceptual model to eye movements, the HGF gives rise to trial-by-trial estimates of prediction errors (ε_2_), volatility beliefs (μ_3_) and learning rates (α), which reflect each participant’s dynamic learning process. We subsequently conducted robust regression analyses (due to the heavy-tailed distributions of the HGF parameters) to examine the relationship between pupil dilation as an index of noradrenergic signalling and:

1. μ_2_, beliefs about *p(normal)*
2. μ_3_, perceived volatility
3. ε_2_, the precision-weighted prediction error about ball bounce trajectory
4. α, the rate of belief updating about *p(normal)*

**Figure 5:**
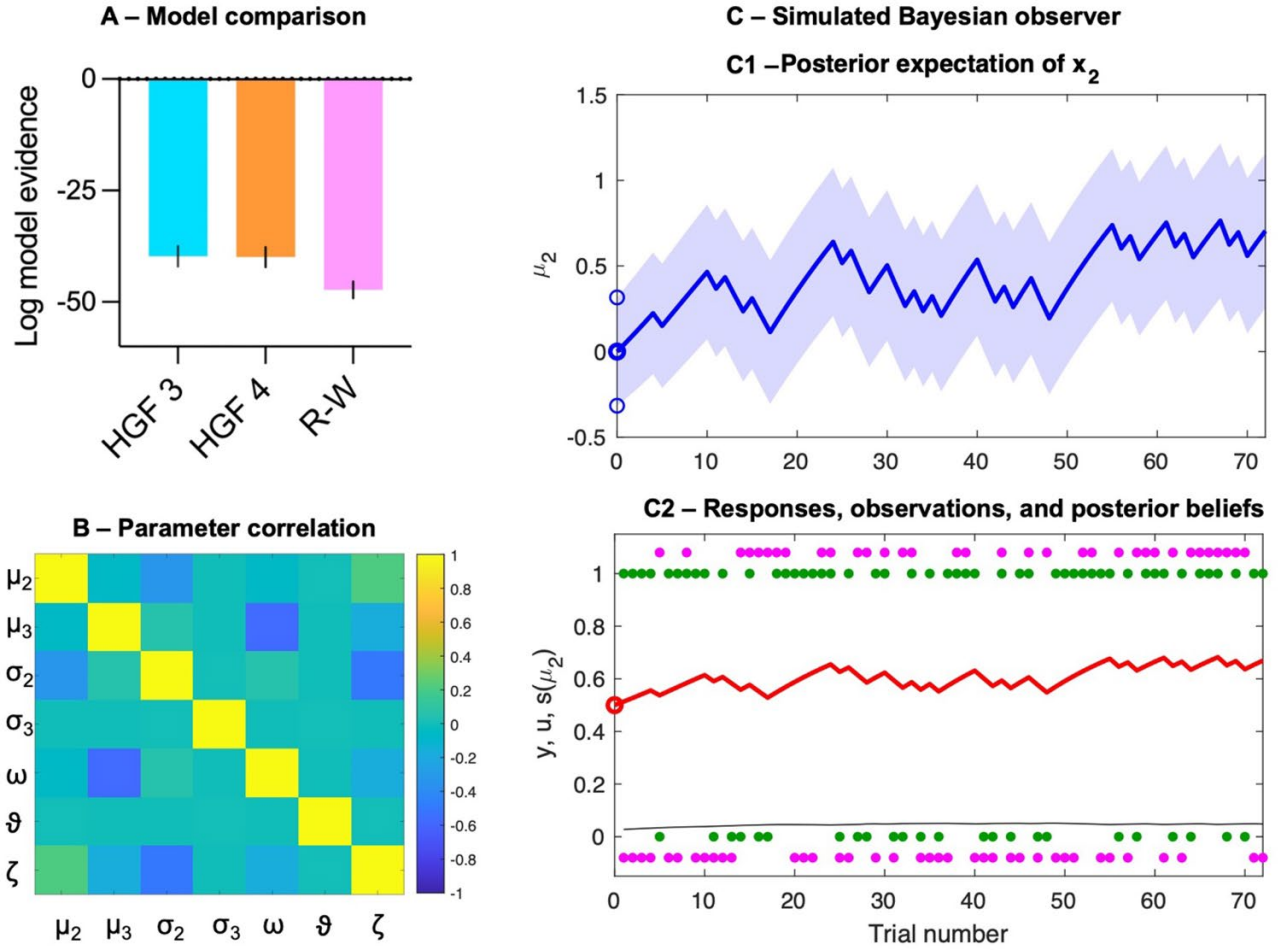
Model development. (**A**) Comparison of model fits showing LME for each model type (and SEM error bars), where higher values indicate better fit. (**B**) Heat plot of averaged correlation matrices for model parameters; parameters should not be highly correlated if they are independently identifiable. No correlations were sufficiently strong as to suggest that one parameter could be substituted for another (σ_i_ = precision of belief at level i; μ_i_ = mean of belief at level i; ω = variance of random walk at x_2_; ϑ = variance of random walk at x_3_; ζ = decision noise). (**C**) Illustration of the belief trajectory for a simulated Bayesian agent in this task. C1 shows the evolving mean and variance of the posterior beliefs for μ_2_. C2 shows inputs/observations (green dots), responses (cyan dots), learning rate (black line), and posterior belief (red line).

The resultant β weights provided an estimate of how the computationally derived metrics of surprise were encoded in pupil size, such that positive β weights indicate pupil size increases alongside prediction errors, increased volatility estimates, or learning rates. This approach followed that of previous work examining the correspondence between pupil dilation and computational models^16,22^. The same β weights were also calculated for the parameters derived from the simulated Bayesian observer (see illustration of simulated belief trajectory in Fig. 5C).

### Statistical analysis

To address our first hypothesis, that anticipatory eye movements would be best explained by a hierarchical Bayesian model, we compared which learning model exhibited the best fit to the data. To do this we compared the log-model evidence (LME) between the competing models (which should be higher in models that better account for the data generating process) using a Bayes factor. After fitting the models, the parameters of interest (μ_2,_ μ_3_, ε_2_, and α) were extracted and a series of robust linear regression analyses were run to obtain individual β-weights for the relationship between model parameters and pupil dilation. The resulting β values were Windsorized by replacing outlying values (> 3.29 standard deviations from the mean) with a score 1% greater or smaller than the next most extreme value. To address hypotheses two (pupil responses would be related to prediction errors and learning rates) and three (pupil responses would be related to perceived volatility), we then assessed whether β weights differed from zero using one-sample *t*-tests for each of the variables of interest. Finally, to address hypothesis four (pupil responses would more closely track personalised models than a theoretical observer model) we generated the simulated behaviour of an optimal Bayesian observer and calculated β weights for each participants pupil response with this theoretical model. We then assessed whether β weights differed from zero, using one-sample t-tests, and also compared the β weights obtained from the personalised models with those from the optimal observer model. Bayes factors were calculated using JASP^55^ to aid the interpretation of any null effects. We interpret BF_10_ > 3 as moderate evidence for the alternative model, and BF_10_ > 10 as strong evidence, with BF_10_ < 0.33 as moderate evidence for the null and BF_10_ < 0.1 as strong evidence for the null. MATLAB code for all data processing is available online (https://osf.io/z96q8/).

## Results

### Model fitting and comparison (H_1_)

Following model fitting (and checks for parameter identifiability—see Fig. 5B), which used a quasi-Newton optimization algorithm^56^, the best model was selected based on the LME for each model type (i.e., *p(data|model)*)). The LME trades-off the accuracy against the complexity of the model (see Fig. 5A). For ease of comparison, a Bayes factor can be computed from the LME by taking an exponential of the difference between two competing models. The rationale for the starting priors chosen for each model is outlined in the supplementary files (https://osf.io/z96q8/).

In support of our first hypothesis, model fits strongly favoured both HGF models over the R-W learning model. Bayes factors showed the data to be considerably more likely under the 3-level (BF = 2048.9) and 4-level (BF = 1662.7) HGF than the R-W model. LMEs were very similar for the three and four level HGFs, with the Bayes Factor marginally favouring the 3-level model (BF = 1.2). Given this was also the simpler structure it was chosen as the winning model. The better fit of the HGF models supports H_1_ and indicates that participants adjusted their eye movements according to principles of hierarchical inference. There has been little work modelling active inference in complex and dynamic real-world tasks, so this initial stage of work itself provides evidence for active inference formulations of perceptual learning and action behaviours.

### Relationships between pupil dilation and model parameters (H_2_–H_4_)

#### Personalised learning models

**Figure 6:**
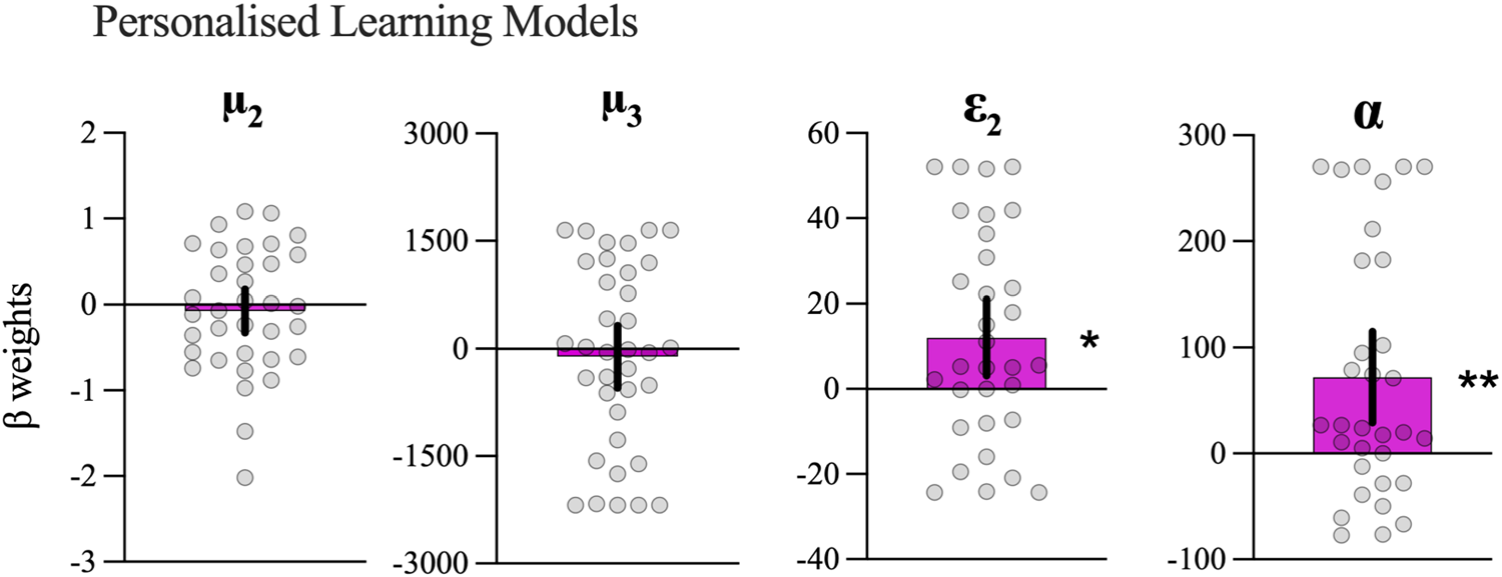
β weights for personalised learning models. Means and 95%CIs for beta weights for each parameter from the active inference models individually fit to each participant. *p < 0.05, **p < 0.01.

To address our hypotheses that pupil responses would be related to both precision-weighted prediction errors (and therefore learning rates; H_2_) and perceived volatility (H_3_), one sample t-tests were run on the β weights derived from the regression analyses to determine whether coefficients were significantly different from zero (see Fig. 6). β weights did not differ significantly from zero for either μ_2_ [*t*(34) =  − 0.61, *p* = 0.55, *d* = 0.10, BF_10_ = 0.21] or μ_3_ [*t*(35) =  − 0.52, *p* = 0.60, *d* = 0.09, BF_10_ = 0.20] parameters, indicating that, for most participants, the task-evoked pupil response did not track beliefs about *p(normal)* or volatility. β weights were, however, positive and significantly different from zero for ε_2_ [*t*(31) = 2.74, *p* = 0.01, *d* = 0.49, BF_10_ = 4.41] and α [*t*(31) = 3.43, *p* = 0.002, *d* = 0.61, BF_10_ = 20.07]. This indicated that pupil dilation tracked the surprise of observations (ε_2_) and the rate of belief updating (α), consistent with the proposed link between pupil dilation and prediction error signalling by the locus coeruleus-norepinephrine system.

#### Simulated Bayesian inference

**Figure 7:**
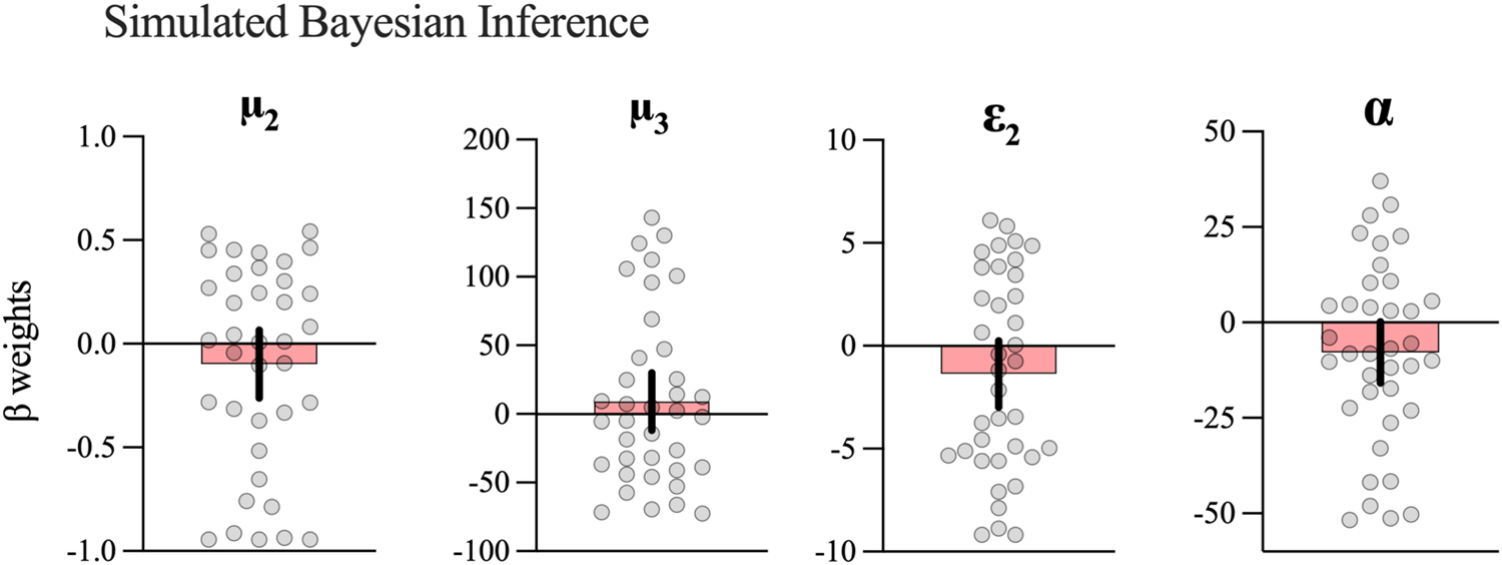
β weights for simulated Bayesian inference. Means and 95%CIs for beta weights for each parameter from the simulated Bayes optimal observer model (Sim).

For the simulated Bayesian agent, the same starting parameters were used (see Table 1) to simulate optimal belief updating over time, given the observed inputs. One-sample t-tests were then run on the β weights, to test whether the pupil response also tracked *theoretical* estimates of prediction errors and volatility (i.e., H_4_). β weights for μ_2_, [*t*(36) =  − 1.22, *p* = 0.23, *d* = 0.20, BF_10_ = 0.35], μ_3_ [*t*(36) = 0.89, *p* = 0.38, *d* = 0.15, BF_10_ = 0.26], ε_2_ [*t*(36) =  − 1.72, *p* = 0.09, *d* = 0.28, BF_10_ = 0.67] and α [*t*(36) =  − 1.99, *p* = 0.054, *d* = 0.33, BF_10_ = 1.03] were not significantly different from zero (see Fig. 7), although α was close to the significance threshold. These results suggest that participants’ pupil response did not track theoretical estimates of precision-weighted prediction errors (ε_2_) or learning rate (α) as they did for the personalised estimates.

**Table 1:** Prior means and variances of the perceptual models.

	Prior mean*	Prior variance
**3-level HGF**		
κ**	1	0
ω	− 5.6	8
ϑ	− 4	0
μ_2_	0	8
σ_2_	0.1	1
μ_3_	1	8
σ3	1	1
**4-level HGF**		
κ**	1	0
ω	− 5.6	8
ϑ	− 4	0
μ_2_	0	8
σ_2_	0.1	1
μ_3_	1	8
σ3	1	1
μ_3_	1	8
σ3	1	1
**R-W model**		
α	0.5	1
*v*	0.5	1

*The HGF class prior means were determined by running a Bayes-optimal simulation of the task (where the variances were set wide to account for individual differences) and taking the resultant posterior means as starting values here (Mathys et al., 2011)^47^. **Kappa, which allows a variable strength of coupling between levels, was fixed to reduce model complexity in light of the relatively few trials.

**Figure 8:**
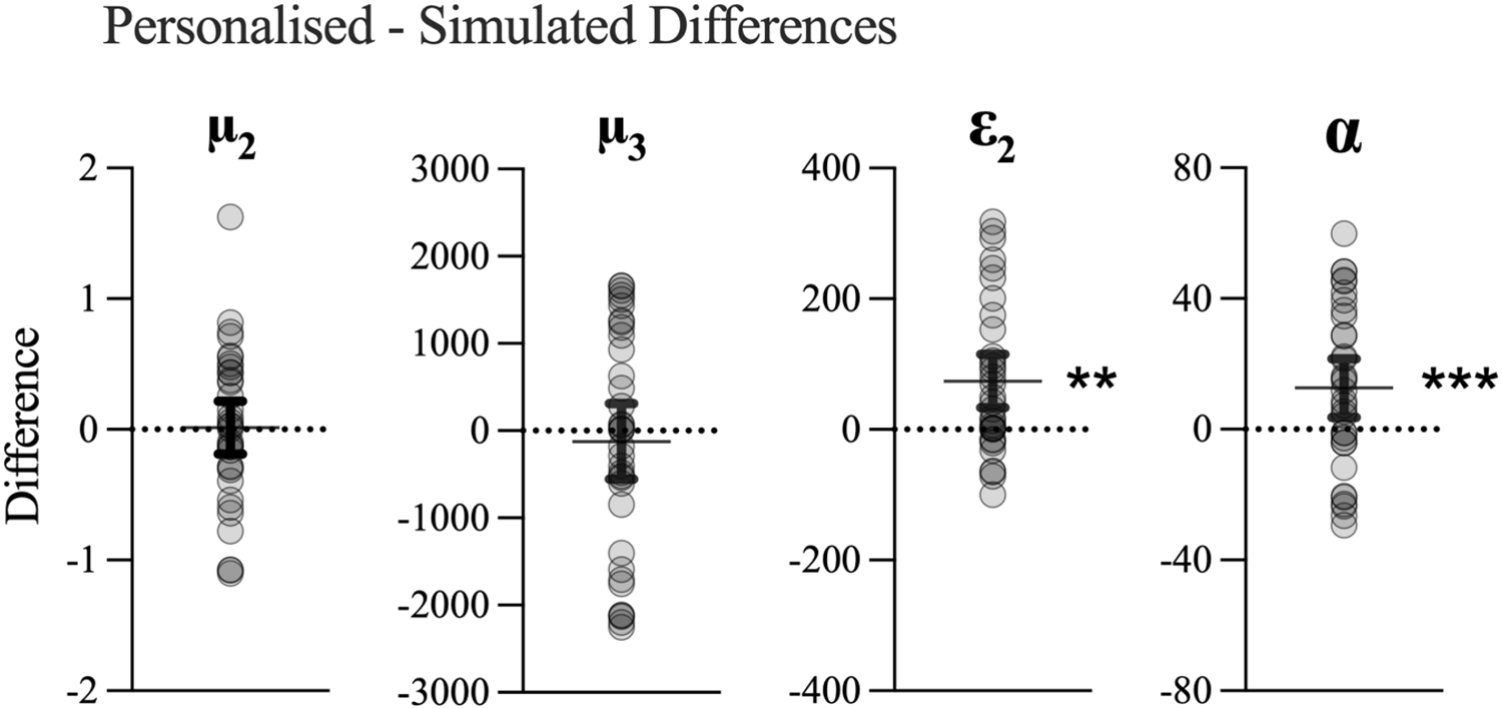
Differences in β coefficients between personalised and simulated models. Figure shows individual data points with means and 95%CIs. **p < 0.01, **p < 0.001.

To confirm whether coefficients were indeed higher for the personalised models (H_4_), we used paired t-tests to compare the beta weights derived from the personalised learning models with the simulated inference models (see Fig. 8). There were no differences for μ_2_ [*t*(34) = 0.15, *p* = 0.88, *d* = 0.03, BF_10_ = 0.18] or μ_3_ [*t*(35) =  − 0.57, *p* = 0.57, *d* = 0.10, BF_10_ = 0.21]. Significant differences were observed for ε_2_ [*t*(31) = 2.93, *p* = 0.006, *d* = 0.52, BF_10_ = 6.58] and α [*t*(31) = 3.76, *p* < 0.001, *d* = 0.66, BF_10_ = 44.02].

## Discussion

In this study, we examined the relationship between physiological encoding of surprise and active inference behaviours during a naturalistic visuomotor task. This work provides an important test of foundational models of the perceptual system and extends current understanding into more realistic human movement skills. Active inference accounts of perception and action propose that action learning should be driven by surprising events that deviate from the agent’s generative model^7,12^. Updates to anticipatory eye movements in our interception task should, therefore, track physiological signalling of surprise^15,21,25,57^. Consistent with these theoretical predictions, estimates of precision-weighted prediction errors (ε_2_) and learning rates (α) derived from HGF models were indeed associated with pupillary signalling of surprise. In contrast to previous work^16,26^, however, we found no evidence for a relationship with volatility beliefs (μ_3_). This work sheds light on the neurocomputational mechanisms underlying perception and action, and thus provides an important empirical test of active inference theory within more naturalistic and dynamic behavioural domains.

In line with our first hypothesis, we observed that a 3-level HGF model^32^ better accounted for trial-to-trial updating of the gaze pitch angle than a simple associative learning model. This finding provides support for active inference accounts of perception and action^6,7^. It is important to note, however, that the better fit of the HGF does not in itself mean eye movements are the result of a Bayesian inference processes in the brain, only that this model better accounted for the data than the alternative learning model. Nonetheless, this result is consistent with a growing body of evidence from this task^35^, and other simpler eye movement tasks that indicate eye movements may follow Bayesian principles^58–60^.

In line with our second hypothesis, surprise-related parameters obtained from the HGF models (ε_2_ and α) were associated with larger task-evoked pupil responses, while beliefs about ball bounciness were not. This finding shows that pupillary signalling of surprise is not related to beliefs as such, but the violation of those beliefs^22^. As predicted by active inference and predictive processing accounts, elevations in surprise signalling also equated to faster belief updating^13,14^. In contrast to previous work^16,26^ and our third hypothesis, we did not observe a relationship between pupil dilation and volatility beliefs. This absence is perhaps understandable, as while the experimental conditions were designed to be volatile, we did not contrast this with clearly distinct periods of low volatility. Additionally, there may have been too few trials to observe relationships between pupil dilation and volatility, which is usually examined over longer trial blocks^26^. Indeed, the estimated values for μ_3_ did not move far from the starting priors for most participants. As subjects learn the value of the mean of a prior distribution within 10–20 trials^61^, our trial numbers were, however, sufficient to observe clear effects for surprise at level-2 of the HGF. As a result, we can be confident that we had sufficient trials for observing effects on precision-weighted prediction errors (ε_2_) and learning rate (α), but we cannot draw definitive conclusions about the absence of a relationship with volatility. Future work should therefore create clearer changes in environmental volatility to further test this relationship.

Despite observing significant β weights for ε_2_ and α parameters, it is important to note that many of these values were still close to zero or even negative, illustrating that these effects were certainly not present in all participants. There are several reasons why this may have been the case. Firstly, as addressed above, we used fewer trials than in most previous work, to ensure that task engagement was maintained throughout the experiment. As a result, people may not have acquired such strong beliefs about the statistics of the environment and therefore experienced dampened surprise responses. Secondly, we used a more naturalistic but less controlled task to examine active inference. Previous work has focused on very simple tasks, such as learning whether an auditory tone is associated with an image of a house or face. By contrast, a significant element of our task involved coordinating a movement response, in addition to implicitly learning about bounce trajectories. The preparation and execution of a motor response is also linked to changes in pupil dilation^62^, so variation in movement kinematics could also have affected (and added variability to) the task-evoked response. Supplementary analyses (see: https://osf.io/e3qcu) indicated that there was a ~ 50 ms difference in swing onset between the two ball types (*p* = 0.003), but as we used peak dilations it is unlikely that this influenced results. There was also some between-participant variation in swing onset times. It was not possible to time-lock recording windows to swing onset as curtailed windows in participants with earlier swings may have prevented the full dilation being detected. Plots of individual pupil traces (see supplementary files: https://osf.io/ws26q) indicated, however, that the time course of the dilation was not heavily influence by swing onset time. Finally, and perhaps most importantly, eye movements are inherently noisy, and the pitch angle measure is not a direct mapping from beliefs to decisions (as may be the case in forced-choice behavioural tasks). Therefore, there is likely to be considerable noise and uncertainty in the mapping of actions to beliefs which would have weakened the relationship we could detect. Given these ambiguities, future research could seek to develop new empirical paradigms that maintain the environmental realism of complex movement skills but seek reduced noise in the mapping of beliefs to action responses.

Unlike the personalised HGF models of anticipatory eye movements, we did not observe any relationship between pupil responses and theoretical estimates of ‘surprise’ derived from an ideal Bayesian observer model. The limited trial numbers may partly account for the lack of relationship with the simulated model, as similar effects have been reported before^21^. This result, however, also supports our assumption that it is important to study the personalised learning process rather than assuming all participants experience the same events as ‘expected’ and ‘unexpected’.

As our results point to the relevance of pupil dilation for understanding physiological signalling of surprise during visually guided actions, future work could use pupil metrics to examine how the encoding of statistical regularities in the environment shapes complex movement skills. For instance, in sports like cricket and baseball, the batter not only makes predictions about the trajectory of the ball in flight^63^, they also weigh up prior contextual information about the most likely speeds, spin, and swing of deliveries that particular bowlers/pitchers might provide^64,65^. Further to this, the relative probabilities of those different balls, and the certainty with which the batter knows them, may further affect control of interceptive movements^66,67^. Therefore, measuring indices of surprise may help to answer questions about how visually guided movements are controlled and the utility of predictive processing and active inference theories for understanding perception and action.

## Conclusion

In summary, this work provides new insights into the control of anticipatory eye movements during complex movement tasks. The results show that that phasic physiological signalling of surprise is a potentially important mechanism in active inference and human sensorimotor behaviour. This work, therefore, serves as a valuable empirical test of increasingly prominent theoretical ideas that fall under the Predictive Processing Framework. It also supports the use of active inference as a framework for understanding the learning and dynamic adjustment of visually guided actions and indicates that future motor learning studies should carefully consider the role of ‘surprise’ in how actions are regulated over time.

## Data Availability

All relevant data and code are available online from: https://osf.io/z96q8/.
